# Effect of Low-Pressure Plasma Treatment Parameters on Wrinkle Features

**DOI:** 10.3390/ma13173852

**Published:** 2020-09-01

**Authors:** Bongjun Gu, Dongwook Ko, Sungjin Jo, Dong Choon Hyun, Hyeon-Ju Oh, Jongbok Kim

**Affiliations:** 1 Department of Materials Science and Engineering, Kumoh National Institute of Technology, Gumi, Gyeongbuk 39177, Korea; bgid90ga@gmail.com (B.G.); duko1293@gmail.com (D.K.); 2 Department of Energy Engineering Convergence, Kumoh National Institute of Technology, Gumi, Gyeongbuk 39177, Korea; 3 School of Architectural, Civil, Environmental, and Energy Engineering, Kyungpook National University, Daegu 41566, Korea; sungjin@knu.ac.kr; 4 Department of Polymer Science and Engineering, Kyungpook National University, Daegu 41566, Korea; dong.hyun@knu.ac.kr; 5 Advanced Materials Research Center, Kumoh National Institute of Technology, Gumi, Gyeongbuk 39177, Korea

**Keywords:** wrinkles, physical structures, plasma treatment, stress relaxation

## Abstract

Wrinkles attract significant attention due to their ability to enhance the mechanical and optical characteristics of various optoelectronic devices. We report the effect of the plasma gas type, power, flow rate, and treatment time on the wrinkle features. When an optical adhesive was treated using a low-pressure plasma of oxygen, argon, and nitrogen, the oxygen and argon plasma generated wrinkles with the lowest and highest wavelengths, respectively. The increase in the power of the nitrogen and oxygen plasma increased the wavelengths and heights of the wrinkles; however, the increase in the power of the argon plasma increased the wavelengths and decreased the heights of the wrinkles. Argon molecules are heavier and smaller than nitrogen and oxygen molecules that have similar weights and sizes; moreover, the argon plasma comprises positive ions while the oxygen and nitrogen plasma comprise negative ions. This resulted in differences in the wrinkle features. It was concluded that a combination of different plasma gases could achieve exclusive control over either the wavelength or the height and allow a thorough analysis of the correlation between the wrinkle features and the characteristics of the electronic devices.

## 1. Introduction

Wrinkles are wavy physical structures with periodicity that can impart flexibility to optoelectronic devices and control the optical path through a material stack. Therefore, they are widely utilized to enhance the properties of optoelectronic devices [1,2,3,4,5,6,7,8]. Rogers et al. reported that the characteristics of transistors and photodetectors can be preserved during device bending by transforming the rigid silicon structure into a wavy wrinkle structure [1]. Bao et al. manufactured stretchable lithium ion batteries with stretchable functional layers by stacking the electrodes and elastic sticky separators on the device-scaled wavy wrinkle structures [2]. In addition to the enhancement of mechanical properties, Takezoe et al. increased the out-coupling efficiency of organic light-emitting devices by sequentially stacking the injection, transport, and active layers on wrinkle structures with high aspect ratios to control the optical path of light [3]. Loo et al. reported that wrinkles can increase the light-harvesting and power conversion efficiencies of organic solar cells by guiding the incident light into the photoactive layer [4].

The primary method of creating wrinkle structures involves pulling the elastomer in one direction, depositing a rigid material thereon, and releasing the stress applied to the elastomer [9,10,11]. Here, stress is induced in the rigid material by the contraction of the elastomer; subsequently, the rigid material generates a wavy wrinkle structure in a direction parallel to the unidirectional stress. Wrinkle structures can be conveniently produced by this method; however, the unidirectional wavy wrinkle structures have no counterparts in the direction perpendicular to the direction of their formation. Therefore, there is no enhancement in the mechanical and optical properties in the direction perpendicular to that of wrinkle formation. To overcome this limitation, the application of biaxial compressive stress by plasma treatment was suggested. The activated ion particles in the plasma collide with a rigid surface and exert stress in all directions of the surface. Plasma treatment creates wrinkle structures with random directionalities that can enhance the mechanical and optical properties in all directions [12,13,14,15].

There are two types of plasma treatment to generate wrinkles, i.e., atmospheric-pressure plasma treatment [16,17] and low-pressure plasma treatment [18,19,20,21]. The advantage of atmospheric-pressure plasma surface treatment is that the plasma generation and surface treatment can be achieved by only applying a high voltage without adjusting the pressure. However, the plasma characteristics depend on the environmental parameters like humidity and temperature, which makes the generation of wrinkles unreliable. Low-pressure plasma treatment can be utilized to achieve environmental control and reliability of the plasma properties and wrinkle features. Here, the sample is placed in a vacuum chamber and the plasma is generated under a low pressure. Time and energy are consumed to create a low-pressure environment for the plasma treatment; however, excellent environmental control is achieved that facilitates the plasma generation and surface treatment. Therefore, low-pressure plasma treatment is widely used in experiments that require precise surface treatment and reliability. Furthermore, atmospheric-pressure plasma treatment allows the generation of plasma only from air. Low-pressure plasma treatment allows the generation of plasma from various gases by blowing the desired types and amounts of gases into the vacuum chamber. This allows a thorough analysis of the variation in the wrinkle features with the plasma characteristics such as the gas type, gas flow rate, power, and treatment time. However, the variation of the wrinkle wavelength with the elastic modulus of the material and its application to electronic devices were the primary subject of studies on wrinkle generation by low-pressure plasma treatment. There is insufficient research on the variation of the wrinkle features with the plasma characteristics.

In this study, we analyzed the variation in the wrinkle features with the parameters of low-pressure plasma treatment such as plasma gas type, plasma power, and treatment time, to enhance the control over wrinkle features and broaden the applications of wrinkle structures. The low-pressure plasma treatment on the surface of a polymer with a specific elastic modulus induces the accumulation of stress on the surface. The relaxation of the accumulated stress over time generates the wrinkle structures. Therefore, the relaxation time after plasma treatment was fixed to exclude the effect of the stress relaxation on the wrinkle shape. The influence of plasma gas type on the wrinkle features was investigated with nitrogen, oxygen, and argon. The wrinkle structures generated by the oxygen and nitrogen plasma possessed similar features. However, the argon plasma generated wrinkles with the highest wavelengths and relatively low heights. When the elastic modulus of the polymeric material increased, the wavelengths and heights of the generated wrinkles decreased regardless of gas type. The nitrogen and oxygen gas flow rates significantly influenced the wavelengths and heights of the wrinkles, while the argon gas flow rate primarily affected the height of the wrinkles. The nitrogen and oxygen plasma showed similar effects on the wrinkle features with variation of the plasma power and surface treatment time; however, the effects of the argon plasma were different to those of the nitrogen and oxygen plasma. These differences were attributed to the opposite charges of the argon ions (positive) and the nitrogen and oxygen ions (negative) in the plasma, along with the differences in the molecular sizes and weights.

## 2. Materials and Methods 

### 2.1. Generation of Diverse Wrinkle Structures by Low-Pressure Plasma Treatment

**Figure 1:**
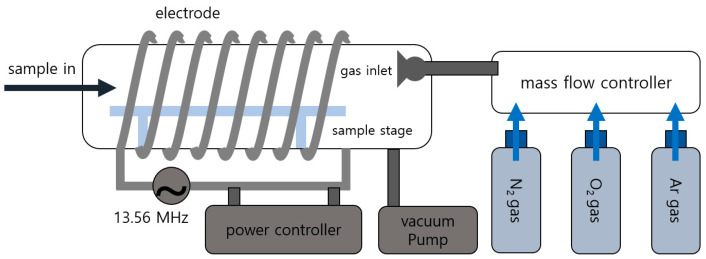
A schematic diagram of a low-pressure plasma treatment system to generate various wrinkle structures.

**Figure 2:**
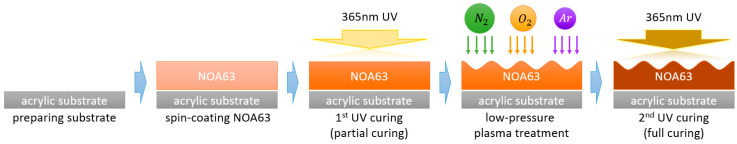
A schematic of the procedure to generate diverse wrinkle structures by low-pressure plasma treatment. The plasma was produced from nitrogen, oxygen, and argon gases under a low pressure.

Figure 1 and Figure 2 show schematic diagrams to describe the low-pressure plasma treatment system and the procedure to generate wrinkles by low-pressure plasma treatment. A 2-mm-thick acrylic plate and Norland Optical Adhesive 63 (NOA 63, Norland Products Inc., Cranbury, NJ, USA) were utilized to prepare the substrate and an ultraviolet (UV)-curable polymer, respectively. NOA 63 was coated on the acrylic plate at 3000 rpm for 30 s after removing the protective film on the acrylic plate. The first stage of curing was performed in a UV curing machine (λ = 365 nm; intensity = 40 W). The total curing energy of the first UV curing step was 1.0–1.6 J, which was sufficient to control the elastic modulus of the UV-curable adhesive material. The NOA 63-coated acrylic plate was placed in a vacuum chamber after the first stage of curing. Nitrogen, oxygen, or argon was blown into the vacuum chamber when the pressure in the chamber reached approximately 3 × 10^−2^ Torr. The gas flow rates were 4–16 sccm. The working pressure depending on gas flow rate is summarized in Table 1. A radio frequency (RF) power source (power = 15–60 W, 13.56 MHz, RF Power Tech, Anyang, Korea) was utilized to generate the plasma of the injected gas. The plasma treatment time was 10–90 s. The plasma-treated sample was taken out of the low-pressure chamber to complete the UV curing (λ = 365 nm) after a relaxation time of 10 min.

### 2.2. Characterization of the Surface Features by Optical Microscopy and Atomic Force Microscopy

The generated wrinkle structures were examined by optical microscopy (OM; BX 51, Olympus, Tokyo, Japan) with a magnification of 500×. The wavelengths of the well-formed wrinkle structures, which were generated by the low-pressure plasma treatment, were measured using the i-Solution Lite program (Olympus). Since the heights of the wrinkles could not be observed by OM, they were analyzed by atomic force microscopy (AFM; NX 10, Park Systems, Suwon, Korea) with a scan range of 45 µm × 45 µm.

## 3. Results

**Figure 3:**
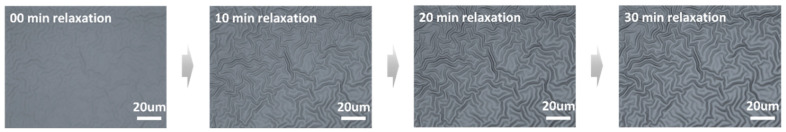
Variation of the surface features of the wrinkles with the relaxation time after the low-pressure plasma treatment. Oxygen gas was used to generate low-pressure plasma. The plasma power and treatment time were 30 W and 30 s, respectively. Gas flow rate was 24 sccm.

Plasma treatment on the surface of a UV-curable polymer forms a rigid layer on top of the soft foundation. This creates a two-layer system with the properties of the soft layer dependent on the first UV curing step and that of the rigid layer dependent on the plasma treatment. During continuous plasma treatment of the two-layer system, the collision of plasma ions induces the accumulation of stress. The relaxation of this accumulated stress generates physical structures like wrinkles. Therefore, the shape of the wrinkles depends not on the magnitude of the accumulated stress but on the magnitude of the stress relieved. Figure 3 shows the morphological evolution of the two-layer system that was dependent on the stress relaxation time after taking out the plasma-treated sample from the low-pressure chamber. A wrinkle was found on the surface of the UV-curable optical adhesive polymer immediately after taking it out of the chamber; however, its height was very low. This indicated that the stress was not completely relieved. The clarity of the wrinkle structure increased after 30 min of relaxation. Therefore, the effect of the plasma treatment conditions on the wrinkle shape should be studied after a fixed relaxation time to exclude the effects of the relaxation time on the wrinkle shape.

**Figure 4:**
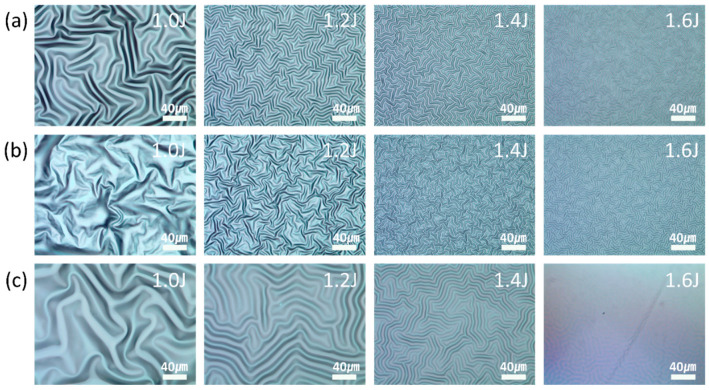
Optical microscopy (OM) images of the various wrinkles that were generated on the ultraviolet (UV)-curable optical adhesive (Norland Optical Adhesive 63; NOA63) at different magnitudes of the first curing energy after (**a**) nitrogen, (**b**) oxygen, and (**c**) argon plasma treatments.

**Figure 5:**
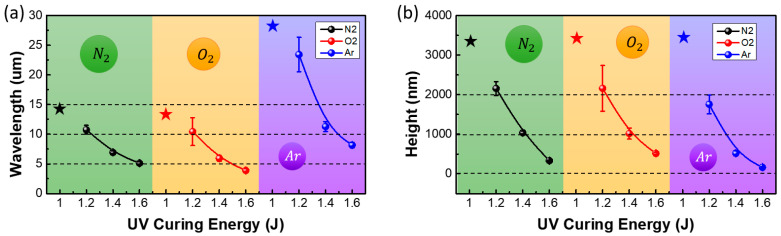
Variation of the (**a**) wavelengths and (**b**) heights of the wrinkles with the first curing energy of the UV-curable optical adhesive (NOA63). Nitrogen, oxygen, and argon were utilized to produce the low-pressure plasma. The asterisks indicate the generation of folds.

To investigate the effect of the plasma gas type on the wrinkle features, nitrogen, oxygen, and argon were utilized as the plasma sources for the plasma treatment on the surface of the UV-curable polymer. The plasma power and treatment time were fixed at 30 W and 30 s, respectively. The gas flow rate was 8 sccm. All samples were fully cured after 10 min of relaxation. Figure 4 shows the OM images of the structures produced on the surface of the NOA 63 polymer when the partially cured polymer was treated with nitrogen, oxygen, and argon plasma in a low-pressure environment. It was observed that the argon plasma generated wrinkles with the highest wavelengths in the identical partially cured conditions. The wrinkle-to-fold transition began occurring at a low first curing energy of 1.0 J. The shape of the wrinkle was observed in detail by AFM. Figure 5 shows the variation in the wavelengths and heights of the wrinkles with the plasma gas type and the first curing energy. When the first curing energy was 1.4 J, the wavelengths of the wrinkles generated by the nitrogen, oxygen, and argon plasma were 6.94 ± 0.28 µm, 5.94 ± 0.5 µm, and 11.3 ± 0.81 µm, respectively. When a partially cured polymer is plasma-treated, the plasma gas penetrates the polymer surface to form a rigid layer. It is known that the argon molecules are heavier than the oxygen and nitrogen molecules that have similar weights. Because argon molecules have a larger mass and, thus, a higher floating potential under the same electron temperature, the energy of argon ions bombarding the polymer surface is higher, leading to deeper penetration into the polymer surface and resulting in a thicker rigid layer [22,23,24]. The wavelength of the wrinkles (*λ_w_*) is proportional to the height of the rigid layer, as shown by the following equation [4]:

(1) $$\lambda_{w}=2\pi h{\left(\frac{E_{f}}{3E_{s}}\right)}^{\frac{1}{3}},$$

where *h* and $E_{f}$ are the thickness and elastic modulus of the rigid layer, respectively. $E_{s}$ is the elastic modulus of the partially cured polymer. Because the thickness of the rigid layer formed by the argon plasma is higher than that of the rigid layers formed by the oxygen and nitrogen plasma, the argon plasma treatment generated wrinkles with the highest wavelengths. Although the weights of oxygen and nitrogen are similar, the wavelength of the wrinkles generated by the oxygen plasma was slightly lower than that of the wrinkles generated by the nitrogen plasma. It was speculated that the oxidizing ability of oxygen, which reacted with the UV-curable polymer, hindered the formation of a rigid layer that was as thick as that formed by nitrogen. The increase in the first curing energy of the polymer decreased the wavelength of the wrinkles generated by all the plasma gases. The first curing energy controls the elastic modulus of the soft polymer layer in the two-layer system that comprises a rigid layer and soft polymer layer. The increase in the first curing energy hardens the UV-curable polymer and increases the elastic modulus of the soft supporting layer. Equation (1) shows that the elastic modulus of the partially cured polymer is inversely proportional to the wavelength of the wrinkles. Therefore, the wavelength decreases with an increase in the first curing energy, regardless of the plasma gas type. The gas type and curing energy for low-pressure plasma treatment can be varied to obtain wrinkles with diverse wavelengths of 3–25 µm to cater to different applications.

**Figure 6:**
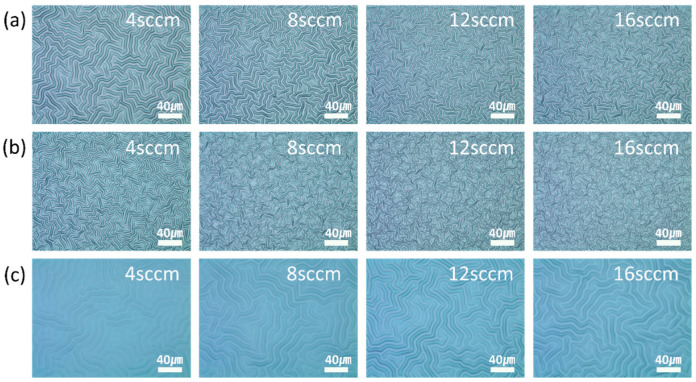
OM images of the wrinkles generated at different gas flow rates. (**a**) Nitrogen, (**b**) oxygen, and (**c**) argon plasma treatments were used to generate these wrinkles.

**Figure 7:**
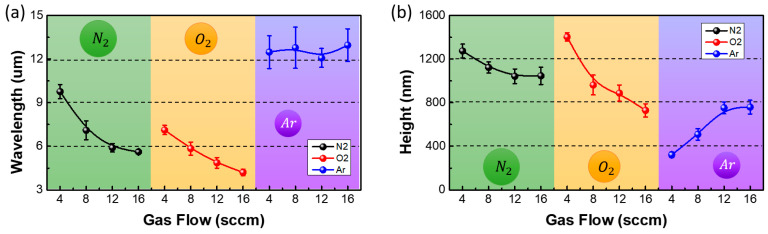
Variation in the (**a**) wavelengths and (**b**) heights of the wrinkles with the gas flow rates during nitrogen, oxygen, and argon plasma treatments.

The variation of the wrinkle features with the plasma gas flow rates was analyzed. Figure 6 shows the OM images of the wrinkles that were generated by increasing the flow rates of nitrogen, oxygen, and argon when the first curing energy of the UV-curable polymer was 1.4 J. The nitrogen and oxygen plasma produced clear images of the wrinkles. Since the argon plasma generated wrinkles with low aspect ratios, the clarity of the images was also low. The wavelengths and heights of the wrinkles were analyzed in detail by AFM. Figure 7 shows the variation in the wavelengths and heights of the wrinkles with the flow rates of nitrogen, oxygen, and argon. As expected from the OM images, the nitrogen and oxygen plasma treatments generated wrinkles with high aspect ratios of 0.13 or more. However, the argon plasma treatment generated wrinkles with low aspect ratios of 0.06 or less. Specifically, the change of the nitrogen flow rate from 4 sccm to 16 sccm resulted in the change of the wavelength from 9.76 µm to 5.62 µm and the height from 1.27 µm to 1.04 µm, showing an aspect ratio above 0.13. When the flow rate of the oxygen plasma was varied from 4 sccm to 16 sccm, the wavelength and height of the wrinkles decreased from 7.12 µm to 4.2 µm and 1.4 µm to 0.73 µm, respectively, while the aspect ratios were 0.16–0.20. The wavelength of the wrinkles produced by the argon plasma treatment remained approximately 12.5 µm, regardless of the gas flow rate. The height and aspect ratio of the wrinkles increased from 0.3 µm to 0.76 µm and 0.03 to 0.06, respectively. However, the aspect ratio of these wrinkles was consistently lower than that of the wrinkles generated by the oxygen and nitrogen plasma treatments. The increase in the gas flow rate increased the plasma density. Since multiple ions simultaneously hit the surface of the UV-curable polymer, the entire surface rapidly became rigid. This decreased the penetration depth of the ions. Therefore, the height of the rigid layer in the two-layer system was low. Since the wavelength of the wrinkles is inversely proportional to the height of the rigid layer, the increase in the gas flow rates of the nitrogen and oxygen plasma decreased the wavelength of the wrinkles. Thus, fine wrinkles with low heights were generated. However, the argon plasma treatment generated wrinkles with similar wavelengths, regardless of the flow rate. Since the argon molecule is smaller than the nitrogen and oxygen molecules, a sufficient distance exists between the argon ions in the plasma, even when the flow rate increases. This allowed the ions in the argon plasma to penetrate to a consistent depth, regardless of the gas flow rate. Therefore, the thickness of the rigid layer and the wavelength of the wrinkles remained relatively unchanged. However, the increase in the density of the argon plasma increased the magnitude of the stress induced in the rigid layer. To release the accumulated stress, the height of the wrinkles increased with the increase in flow rate.

**Figure 8:**
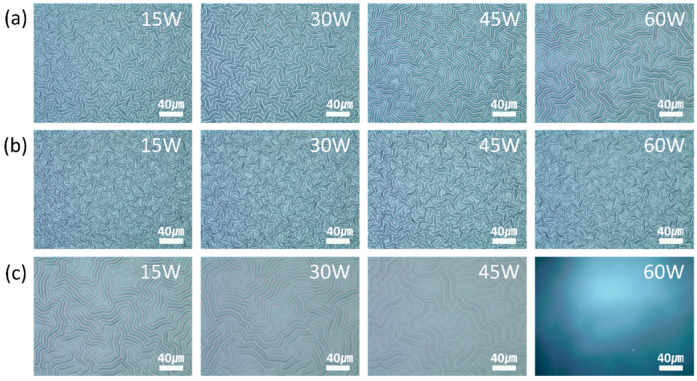
OM images of the various wrinkles generated by (**a**) nitrogen, (**b**) oxygen, and (**c**) argon plasma treatments at different magnitudes of the plasma power.

**Figure 9:**
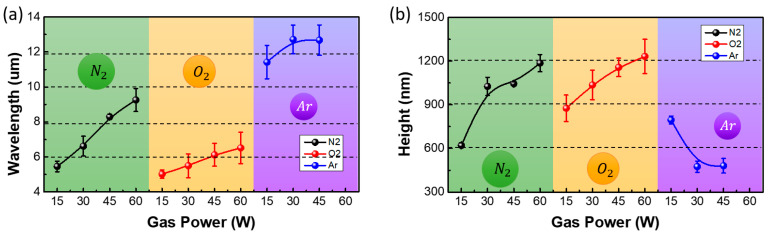
Variation in the (**a**) wavelengths and (**b**) heights of the wrinkles with the plasma power. The wrinkles were generated by nitrogen, oxygen, and argon plasma treatments.

Figure 8 and Figure 9 show the OM images and the variation in the wavelengths and heights of the generated wrinkles at different magnitudes of the plasma power. The wavelengths and heights of the wrinkles generated by the nitrogen and oxygen plasma increased with the increase in plasma power. The increase in plasma power increased the total ion current on the substrate, leading to an increase in the penetration depth of the plasma gas and the thickness of the rigid layer. This resulted in an increase in the wavelength of the wrinkles. The increase in the power of the nitrogen and oxygen plasma increased the accumulated stress in the wrinkles. The wrinkles grew in height to relieve the accumulated stress. However, the increase in the power of the argon plasma increased the wavelength and decreased the height of the generated wrinkles. The argon molecules are smaller and heavier than the nitrogen and oxygen molecules; moreover, the argon plasma has positively charged ions, while the nitrogen and oxygen plasma have negatively charged ions. Therefore, there were differences in the trends of stress accumulation and relaxation. The argon plasma penetrated to a greater depth with an increase in the plasma power. This increased the thickness of the rigid layer; therefore, the wavelength of the wrinkles also increased. The accumulated stress in the wrinkles was relieved locally due to the small argon molecules. Therefore, the height of the wrinkles decreased.

**Figure 10:**
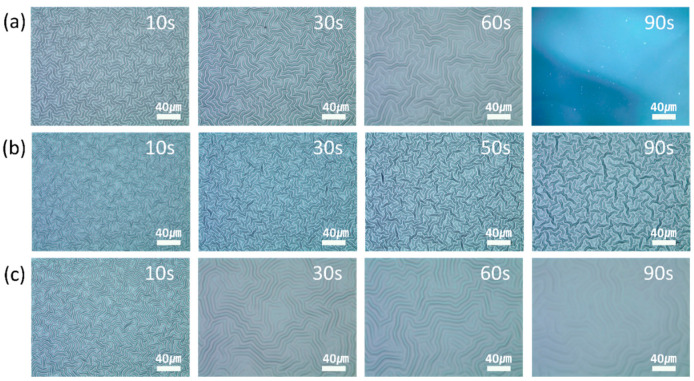
OM images of the wrinkles that were generated after different treatment times. The wrinkles were generated by (**a**) nitrogen, (**b**) oxygen, and (**c**) argon plasma treatments.

**Figure 11:**
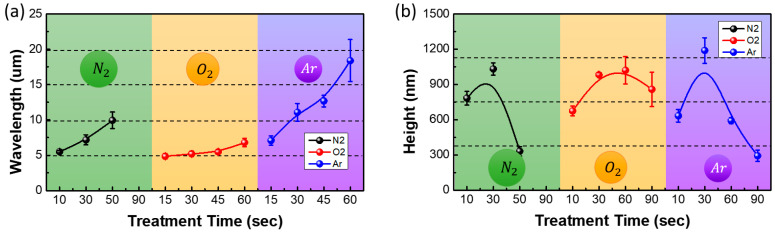
Variation of the (**a**) wavelengths and (**b**) heights of the wrinkles with the treatment times (10–90 s) of the nitrogen, oxygen, and argon plasma treatment.

Figure 10 and Figure 11 show the OM images and the variation in the wavelengths and heights of the wrinkles at different plasma treatment times. The wavelength of the wrinkles increased with the increase in the treatment time, and the wrinkle-to-fold transition began after approximately 90 s of plasma treatment. The accumulated stress in the two-layer system increased with the increase in the treatment time. Therefore, the wavelength of the wrinkles increased. Eventually, the accumulated stress increased beyond a threshold value and a deep fold was formed to release the accumulated stress. The wrinkle-to-fold transition that was caused by the increase in the plasma treatment time also affected the height of the wrinkle. At a plasma power of 30 W, the height of the wrinkles, regardless of the plasma gas type, was maximum when the treatment time was 30–60 s and decreased thereafter. Since the accumulated stress in the wrinkles was relieved by the formation of the fold, it decreased after the wrinkle-to-fold transition. Therefore, the height of the wrinkles decreased beyond the critical stress and critical treatment time.

## 4. Conclusions

In this study, the effects of various parameters on the generation of wrinkles by low-pressure plasma treatment were investigated. These parameters included the gas type, first curing energy of the UV-curable polymer, gas flow rate, plasma power, and treatment time. The effect of the plasma gas type on the thickness of the rigid layer produced wrinkles with diverse features. The first curing energy and the gas flow rate affected the wrinkle shape by changing the elastic modulus of the UV-curable polymer and the plasma density, respectively. The argon molecules are heavier and smaller than the nitrogen and oxygen molecules of similar weights and sizes; moreover, the argon plasma comprises positive ions, while the nitrogen and oxygen plasma comprise negative ions. Therefore, the effects of the plasma power and gas flow rate varied with the plasma gas type. These results confirmed that a combination of gases in the plasma could control the wavelength at a constant height or control the height at a constant wavelength. Thus, the diverse applications of electronic devices could be catered to; moreover, it would enable a thorough analysis of the relationship between the wrinkle features and device characteristics.

## Figures and Tables

**Table 1:** Working pressure depending on plasma gas (nitrogen, oxygen, and argon) and its flow rate.

Plasma Gas.	N_2_				O_2_				Ar			
**Flow Rate (sccm)**	4	8	12	16	4	8	12	16	4	8	12	16
**Working Pressure** ($\times 10^{-1}\;\mathrm{Torr}$)	2.1	4.0	6.2 *	8.3 *	2.2	3.6	5.0 *	6.3 *	1.6	2.8	3.7 *	4.6 *

The asterisks (*) indicate the estimated working pressure from the ratio between working pressure at 8 sccm and working pressure at a corresponding gas flow rate in a modified low-pressure plasma chamber.
